# Hospital Expenditure.—The Commissariat

**Published:** 1895-12-14

**Authors:** 


### The Institutional Workshop.

### HOSPITAL EXPENDITURE.—THE COMMISSARIAT.

### IX.—THE NURSES' TABLE.—PROVINCIAL.

Turning now to provincial institutions, no very perceptible change can be observed in the standard or cost of the nurses' board. In few provincial institutions has any very precise idea been formed of the cost of provisioning this class of the inmates, as distinct from the remainder; but the cost of the "household," as distinguished from the patients, is in some cases ascertainable.

At the Derbyshire Royal Infirmary the board of nurses and servants is found to cost 5s. 9d. a week. The diet is on a liberal scale and of excellent quality. The cost of the nurses' board amounts to £5 10s. per occupied bed, or 27 per cent. of the total cost of provisions.

At the Glasgow Western Infirmary the cost of nurses and servants works out at from 5s. to 5s. 9d. a week. In 1893 the cost of boarding the 95 nurses amounted to £3 15s. per occupied bed, or 16 per cent. of the total cost of provisions.

At Newcastle Royal Infirmary the cost of the nurses' board is estimated at 4s. a week, beer not included; this would equal 5s. a week with beer, and the total cost per occupied bed would then be £2 10s. yearly, and the percentage of total cost of provisions 13. An additional meal provided for nurses on night duty adds an extra shilling weekly to the cost of their board.

The following is the diet table for the household of the Sussex County Hospital:—

| | s. | d. |
|---|---|---|
| Daily. | | |
| Meat cooked with bone, 11 oz. (say 14 oz. uncooked), at 4½d. lb. ... ... ... ... | 0 | 4 |
| Bread, 14 oz., at 4¼d. a quartern ... ... | 0 | 1 |
| Potatoes, 8 oz., at 1d. per lb. ... ... ... | 0 | ½ |
| Beer or porter, 1 pint, at 1s. per gallon .. | 0 | 1½ |
| Milk, ¼ pint, at 10d. per gallon ... ... | 0 | ¼ |
| Total daily ... ... ... ... | 0 | 7¼ |
| Or weekly ... ... ... ... | 4 | 2¾ |
| Weekly. | | |
| Butter, ½ lb., at 1s. 2d.... ... ... ... | 0 | 7 |
| Cheese, ½ lb., at 7d. ... ... ... ... | 0 | 3½ |
| Coffee, ½ lb., at 11d., or tea and coffee ... | 0 | 5½ |
| Sugar, ½ lb., at 2½d. ... ... ... ... | 0 | 1¼ |
| Bacon, ½ lb., at 8½d. ... ... ... ... | 0 | 4¼ |
| Eggs, 4, at 1¼d. each ... ... ... ... | 0 | 5 |
| Total weekly cost ... ... ... | 6 | 5¼ |

No allowance is made here for flour, puddings of any kind, fruit, vegetables, or condiments, such as pepper, salt, and vinegar, &c. The total cost can hardly be less than 7s. a week, and inquiries made in different localities have shown this to be about the usual average for the household in fair-sized institutions.

Few hospitals now make these definite allowances for the household. It is beginning to be generally recognised that all appetites cannot be measured by the same rule, and that though among large numbers it will be safe to reckon on a fixed quantity being used per head, yet when the amounts are rigidly assigned to each one some will have far too much if all are to have sufficient.

One more specimen of an allowance table may be quoted; it is the one used in the Belfast Royal and Throne Hospitals for servants, and in the Throne Hospital for nurses also. The allowances are for the week:—

| | s. | d. |
|---|---|---|
| Tea, 3½ oz., at 1s. 6d. per lb. ... ... ... | 0 | 4 |
| Sugar, 1 lb. ... ... ... ... ... | 0 | 2 |
| Bread, 6 lb., at 9s. 3d. per cwt. ... ... | 0 | 6 |
| Potatoes, 14 lb., about ... ... ... ... | 0 | 7 |
| Butter, ½ lb., about ... ... ... ... | 0 | 7 |
| Beef, 1¾ lb., at 7d. per lb. ... ... ... | 1 | 1 |
| Vegetables, twice a week, about ... ... | 0 | 2 |
| Eggs, 6, about ... ... ... ... ... | 0 | 6 |
| Bacon, ¾ lb., at 6d. per lb. ... ... ... | 0 | 4½ |
| Sweet milk, 7 pints, at 9⅞d. per gallon ... | 0 | 8½ |
| Sour milk, 7 pints, about ... ... ... | 0 | 4 |
| | 5 | 4 |

Beer is not reckoned, but if allowed that would make an addition of at least 10½d. per week. The curious disproportion between the allowance of potatoes and meat calls for remark, and, with all due allowance for national customs, four ounces of meat (apparently weighed uncooked) can hardly be considered a satisfactory daily allowance for women engaged in hard work. The large quantity of milk (a quart a day) taken in conjunction with the potatoes would compensate to a certain extent for this deficiency, the buttermilk especially containing a large quantity of the nitrogenous substance casein.

It has been said that about 7s. a week each is a fair average to reckon for the expenses of the household in provincial institutions containing over 50 beds. Unless the housekeeper is exceptionally able, it is hardly possible to provide a liberal diet with due variety, and including malt liquor, for persons in active employment at a cost much under one shilling a day. It can be done well for rather less than this, but it seldom is. Several circumstances, however, may result in modifying the expenditure under this heading in individual hospitals.

In the first place, some provincial hospitals, in which a strong local interest is aroused and maintained, such as the Addenbrooke, at Cambridge, are peculiarly well off as regards gifts in kind. The articles given—fruit, vegetables, and sundry kinds of groceries, often unsuited for invalids—are of just such a nature as to lend welcome variety to the nurses' table, and the additional cost of such extras would, if they were purchased, figure largely in the accounts.

And secondly, in many provincial establishments, the garden is a very real gain, and its expenses are rarely reckoned among the cost of provisions. Compare the outlay for vegetables at York County Hospital (103 occupied beds), £27, with that at the Royal Free (112 occupied beds), £100; or the Great Northern Central (68 occupied beds), £55.

The number of persons to be boarded, only affects very slightly the cost of provisions. One hundred persons do not, it is true, cost a hundred times as much as one; but they will cost fully as much as five times the amount spent on twenty. In reckoning together every item of household expenditure, it can, of course, be mathematically demonstrated that every additional person boarded reduces the average cost of the whole number by at least a fraction of a penny. But when the cost of fuel, service, &c., common to all is omitted, the fraction is hard to determine, and the gradations are imperceptible. A clever matron will probably succeed in procuring more variety for her nurses should their number be increased for any reason, and might succeed in effecting small savings in certain articles, but it is idle to expect an institution boarding 150 nurses to show a smaller average per head for the provisions than one boarding 40 or 50. Indeed, experience points entirely the other way, as readers who have studied the cost of nurses' board at some of the large London hospitals will have perceived. For it is probable that any economy which might result from the convenience of providing for a large number is more than overbalanced by the lack of the individual control which is possible in smaller establishments.

We have not so far touched upon the question of the quality of the provisions, although to some it may appear that this is the most important factor of all in determining expenditure. It is, however, matter of common experience that in places where the food is coarse and badly prepared the cost is fully as high, if not higher than where all is appetising and wholesome. For the same careless management which sanctions the bad fare is nearly always reckless about waste and ignorant about prices.

